# Effect of Aggregate Size and Water/Cement on Compressive Strength and Physiological Performance of Planting Concrete

**DOI:** 10.3390/ma15196685

**Published:** 2022-09-27

**Authors:** Jianguo Chen, Weilian Du, Guanqi Zhao, Mingsheng Shi, Binghan Xue

**Affiliations:** 1 Yellow River Laboratory, Zhengzhou University, Zhengzhou 450001, China; 2 Guangxi Key Laboratory of Water Engineering Materials and Structures, Guangxi Institute of Water Resources Research, Nanning 530000, China; 3 College of Mechanics and Materials, Hohai University, Nanjing 211106, China

**Keywords:** planting concrete, aggregate properties, pore structure, mechanical properties, physiological properties

## Abstract

Planting concrete, an eco-friendly concrete in which plants can grow directly, has been widely used in roof greening and the slopes of rivers. Porosity and compressive strength are important indicators for evaluating the properties of planting concrete. By preparing planting concrete with different aggregate gradations (10–30 mm, 20–40 mm) and water–cement ratios (0.25, 0.27, 0.29, 0.31, 0.33), the effect of aggregate gradation and water–cement ratio on the porosity and compressive strength of the planting concrete was analyzed, the intrinsic relationship between aggregate gradation and plane pore parameters was studied, the strength growth pattern and microscopic strengthening mechanism were studied, the relationship between porosity and compressive strength of the planting concrete were explored, and a tall fescue planting experiment was carried out to evaluate the plantation performance of the planting concrete. The results show that under the same conditions of water–cement ratio, the smaller the particle size of the aggregate, the smaller the porosity of the plane, and the denser the structure. The average diameter of the planting concrete shows an exponential relationship with the porosity of plane. The early growth of the compressive strength of the planting concrete is rapid; the compressive strength has a linear relationship at the ages of 7 days and 28 days. Compared to polynomial and logarithmic functions, the exponential function gives a better insight into the relationship between the porosity and compressive strength of the planting concrete. Tall fescue seeds germinate and grow well; height, cover, and leaf rootstock and element content of plants can be used as indicators to assess the performance of vegetated concrete planting.

## 1. Introduction

Planting concrete is a porous concrete and its products that can satisfy the requirements of green plants, with continuous internal pores that allow water, air, etc., to pass through easily. It not only guarantees its basic function as a material structure, but also reduces the load on the environment and plays an important role in enhancing moisture and heat exchange in the ground, regulating temperature and humidity, and absorbing noise, with significant eco-benefits [1,2,3,4,5].

Despite the many important benefits of planting concrete, the efficient growth of plants is still influenced by porosity. The greater the porosity of the planting concrete, the more space there is for plant growth and the better the plants will grow; however, higher porosity leads to lower strength for concrete [6]. The type, shape, and size of the coarse aggregates and the water–cement ratio are influencing factors that greatly affect the physical and mechanical properties of the planting concrete, such as porosity and strength [7,8]. Kim et al. [9] replaced the natural coarse aggregate with blast furnace slag, and when the blast furnace slag aggregate was replaced from 0 to 100%, the porosity of the planting concrete increased from 15 to 27.5%. At the same time, the compressive strength decreased from 13 MPa to 11.5 MPa. Furthermore, using recycled aggregates as a substitute for natural aggregates with the same aggregate gradation of 5–25 mm [7], it was found that the compressive strength of the planting concrete decreased by 32%. Compared to natural aggregates, recycled aggregates have undergone mechanical wear and chemical degradation over a longer period of time, and mechanical damage from the crushing process causes more microcracks in the aggregates, resulting in their poorer performance as concrete aggregates; another reason for poor performance is the presence of mortar and interface transition zones in recycled concrete aggregates [10], which leads to weaker bonding between recycled aggregates and cement [11]. Malaiskiene et al. [12] found that the aggregate particle size has a significant effect on the physical and mechanical properties of porous concrete. Liu et al. [13] used two different sizes of aggregate distribution; studies have found that a larger size of the aggregate and a higher effective porosity lead to lower compressive strength. Da Costa et al. [14] found that the compressive strength of concrete depends on the porosity and the bond of the aggregate to the paste and that a suitable compressive strength can be obtained by varying the W/C. The porosity and compressive strength of the planting concrete in practical engineering are still influenced by the type of aggregate, aggregate gradation, water–cement ratio, etc. Based on this, resolving the contradiction between the porosity and strength of planting concrete and then obtaining a balance of the relationship between the two is the key scientific issue in promoting the use of planting concrete.

In this study, planting concrete was prepared with two aggregate gradations and five water–cement ratios, the effect of aggregate size and water–cement ratio on the porosity and compressive strength of planting concrete was studied, the effect of different aggregate sizes on the two-dimensional pore characteristics of planting concrete was explored, and the intrinsic link between planar pore parameters was investigated. This study also explored the relationship between water–cement ratio, porosity, and compressive strength, establishing a relationship equation between porosity and compressive strength. By optimizing the design of the mixing proportions, parameters for a planting concrete formulation to support the growth of tall fescue are proposed. A system of evaluation indicators for plant growth in planting concrete was constructed by testing the growth status, above- and belowground biomass, and the total nitrogen and phosphorus content of tall fescue plants with different planting methods and different sowing densities.

## 2. Raw Materials and Test Methods

### 2.1. Raw Materials

Cement: The test was performed using Hai Luo brand ordinary Portland cement of strength grade 42.5. The chemical composition and physical properties are shown in Table 1 and Table 2, respectively. Aggregates: The continuous gradation of coarse aggregates is not suitable for the formation of pores, and the small size of aggregates will not be conducive to the formation of large pores, while the large size will have a negative impact on the mechanical properties, so it is advisable to use single gradation or broken gradation aggregates for the preparation of planting concrete. Limestone with a dense structure and smooth surface was selected as the aggregates, and the aggregate gradation was selected as 10–30 mm and 20–40 mm; the physical properties of the aggregates are shown in Table 3.

### 2.2. Experimental Method

#### 2.2.1. Mixing Proportions of Planting Concrete

According to literature research, the suitable aggregate–cement ratio of planting concrete is between 3.75 and 5.33, and the water–cement ratios were selected as 0.25, 0.27, 0.29, 0.31, and 0.33 [9,15,16,17]. In this study, the aggregate–cement ratio was selected as 4.90, for a total of 10 sets of planting concrete specimens.

#### 2.2.2. Planting Concrete Preparation

**Figure 1:**
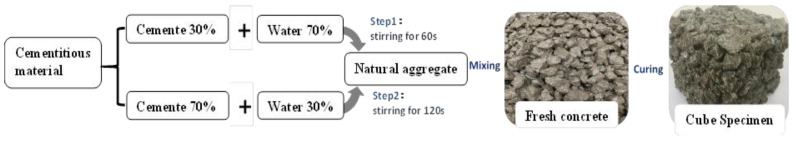
Preparation flow chart.

First, 30% cement, 70% water, and all aggregates were mixed together in a forced single-shaft mixer for 60 s. This process allows the slurry to be fully mixed and evenly wrapped on the surface of the coarse aggregate. Then, 70% cement and 30% water were added and mixed for 120 s until the slurry layer was uniformly wrapped around the surface of the coarse aggregate and had a metallic luster. Finally, the well-mixed concrete mix was loaded into the test mold in three layers, with each layer being 1/3 of the overall test mold volume, and 15 poundings were inserted in sequence from the edge to the inside. After one layer of plugging and pounding was completed, the next layer of molding was carried out, and finally the specimen was manually compressed and the surface of the specimen was repaired and leveled, as shown in Figure 1.

#### 2.2.3. Test Methods

(1) Compressive strength: Compressive strength is an important indicator of the mechanical properties of planting concrete. To test the compressive strength of the planting concrete, cubic samples of planting concrete with dimensions of 150 mm × 150 mm × 150 mm were used [18]. There were three specimens in each group, and a total of 30 specimens were made. After forming and demolding the specimens, the compressive strength tests were carried out in standard holding rooms (temperature 20 ± 2 °C and 99% humidity) until the ages of 7 days and 28 days. The compressive strength is given in Equation (1).

(1) $$f_{c}=\frac{F}{A}$$

where *f_c_* is compressive strength at 7 days and 28 days (MPa), *F* is the breaking load of theplanting concrete sample (N), and *A* is the cross-sectional area of the specimen (mm^2^).

(2) Porosity: The porosity of planting concrete can be classified into connective porosity and gross porosity. The connective porosity is an important indicator for positive plant growth in planting concrete (porosity in the subsequent text refers collectively to connective porosity). The specific test is as follows [19]: After leaving the prepared planting concrete blocks to age for 28 days, the test block mass *W*_1_ was weighed. After the mass was soaked in water for 24 h and saturated with water, its mass in water *W*_2_ was weighed. The connective porosity is given in Equation (2).

(2) $$P_{a}=(1-\frac{W_{1}-W_{2}}{\rho_{W}v})\times 100\%$$

where *P_a_* is the connective porosity of the specimen, *W*_1_ is the mass of the specimen in air after 28 days of curing, *W*_2_ is the mass of the specimen in water after 24 h of immersion, *V* is the volume of the specimen, and *ρ*_w_ is the density of water. The connective porosity of the planting concrete is taken as the average of three specimens.

**Figure 2:**
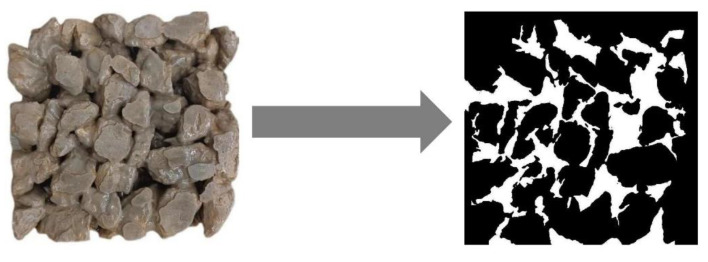

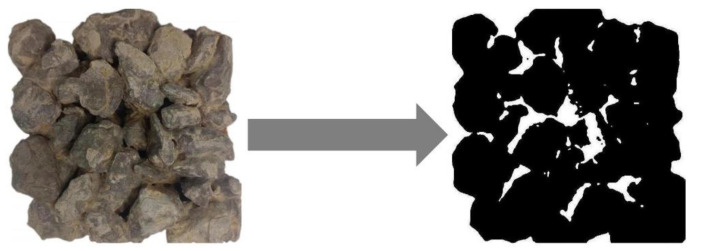
Image processing.

(3) Surface porosity analysis: A rock cutter was used to slice the cubic standard specimen into layers, and the specimen was evenly cut into 5 pieces with a slice thickness of 30 mm. The upper and lower surfaces of the cut section should be parallel and the surface should be smooth. The cut surfaces were polished with 150-grit sandpaper for more than 20 min to eliminate the scratches caused by the cut sections so that the surface of the section was smooth and flat and the structure of the hole was visible. A digital camera was used to photograph the concrete slices, and the images were equalized and grayscale-processed. Combined with the histogram image features, the pore grayscale values were judged to be in the range of 50–80, and the image binarization was achieved using the im2bw function in the mathematical software. The processing result is shown in Figure 2. The white part is the porosity part. After the processing of the slices, they were imported into the Image-Pro Plus 6.0 software (US MEDIA CYBERNETICS, Rockville, MD, USA) and the appropriate parameters were set. The pore structure parameters calculated were pore area, pore diameter (mean), and plane porosity. The plane porosity is calculated as a percentage of the slice area by the pore area and the plane porosity is calculated as in Equation (3):

(3) $$P_{b}=\frac{\sum A_{i}}{A_{t}}$$

where *P_b_* is the porosity of the specimen section plane, *A_i_* is the area of pore number *i*, and *A_t_* is the area of the plane.

**Figure 3:**
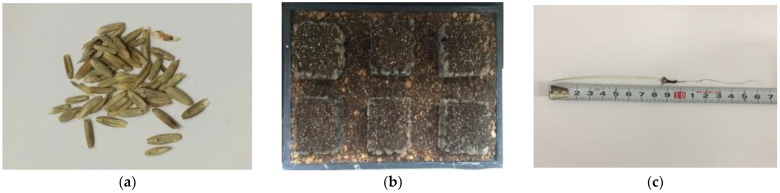
Planting experiment. (**a**) Tall fescue seeds; (**b**) Surface planting structures; (**c**) The measurement of root length.

(4) SEM microscopic experiment: For SEM specimen preparation, concrete specimens of 20–40 mm grade with 0.29 water–cement ratio were cured to 7 days age and broken. A block with a section of about 1 cm × 1 cm × 1 cm was taken and the section was left untreated; the specimen sections were left untreated and then put into anhydrous ethanol for 48 h to end hydration. A small piece of the specimen was glued to the sample table and surface gold plating was performed to increase the electrical conductivity, and the thickness of the gold spray was about 20 nm. The sample was tested by SEM using a Gemini SEM300 scanning electron microscope (Carl Zeiss AG, Oberkochen, Germany) for SEM testing of the sample morphology, and the test was performed using a secondary electron SE2 detector.
(5) Planting experiment: Tall fescue was selected for planting in the experiment, and the seeds are shown in Figure 3a. Tall fescue is a perennial ground cover plant of the grass family and is one of the plants that grow well in a resilient manner; it can adapt to a degree of acidity and salinity, with the most suitable environment for tall fescue growth having the pH of 4.7–8.6. Seeds should be kept refrigerated at 0–10 °C prior to sowing and soaked in water for germination 24 h prior to sowing. The top-set planting configuration used for the experiment is shown in Figure 3b. The root length was measured using a straightedge to determine plant height and root length during the experiment, and the germination and growth of tall fescue in soil and planting concrete were noted. The method of measuring root length is shown in Figure 3c.

(6) Determination of biomass and elemental levels in plant leaves and roots: After 50 days of growth, tall fescue plants grown with different planting methods and planting densities were taken as samples. The tall fescue plants were removed from the planting concrete completely; the stems and leaves were separated from the root system with scissors, and the root system was rinsed with distilled water. Each pot of stem and root samples was separately placed in a 115 °C oven for 15 min to inactivate the enzymes and then dried at 75 °C. Three tall fescue plants were taken for measuring the biomass of stems, leaves, and roots, and another three plants were taken for measuring the total nitrogen and phosphorus content in tall fescue. The total nitrogen content was determined by the H_2_SO_4_–H_2_O_2_ decoction method, and the total phosphorus content was determined by the alkali fusion molybdenum antimony anti-colorimetric method.

## 3. Results and Discussion

### 3.1. Effect of Water–Cement Ratio and Aggregate Particle Size on Porosity

**Figure 4:**
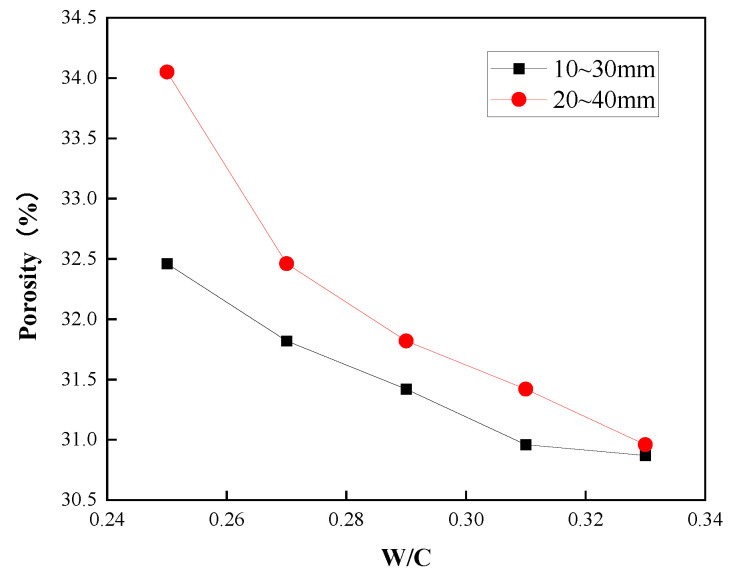
Effect of water–cement ratio and aggregate particle size on porosity.

**Figure 5:**
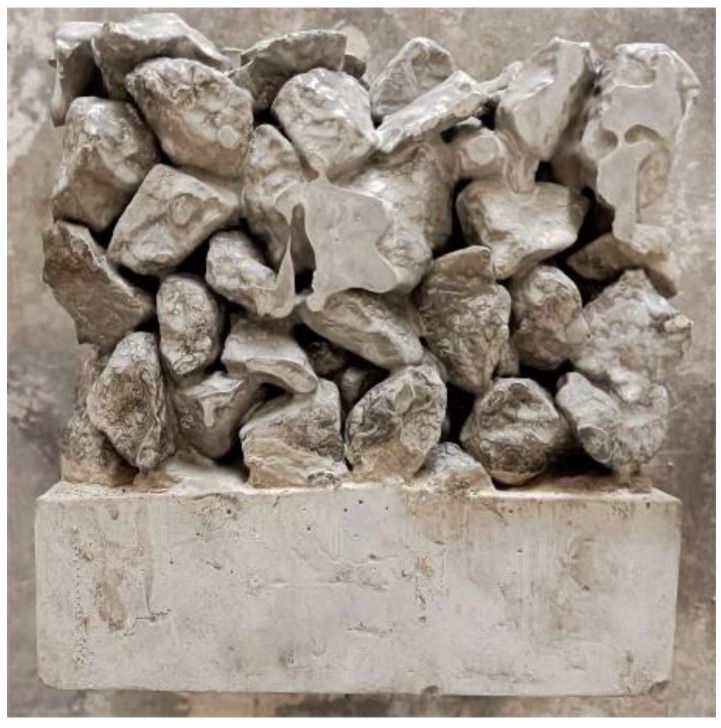
Planting experiment.

The effect of the water–cement ratio on the porosity of the planting concrete is shown in Figure 4. As shown in Figure 4, the porosity of planting concrete decreases as the water–cement ratio increases; larger aggregate particle sizes result in greater porosity at the same water–cement ratio. This is due to the increased fluidity of the slurry as the water–cement ratio increases. In the process of preparing and forming the planting concrete, it is easy to cause the slurry to segregate and accumulate at the bottom of the concrete under the action of gravitational force, causing plugging of holes (as shown in Figure 5), which causes a decrease in porosity.

### 3.2. Characterization of the Pore Structure of Planting Concrete Surfaces

**Figure 6:**
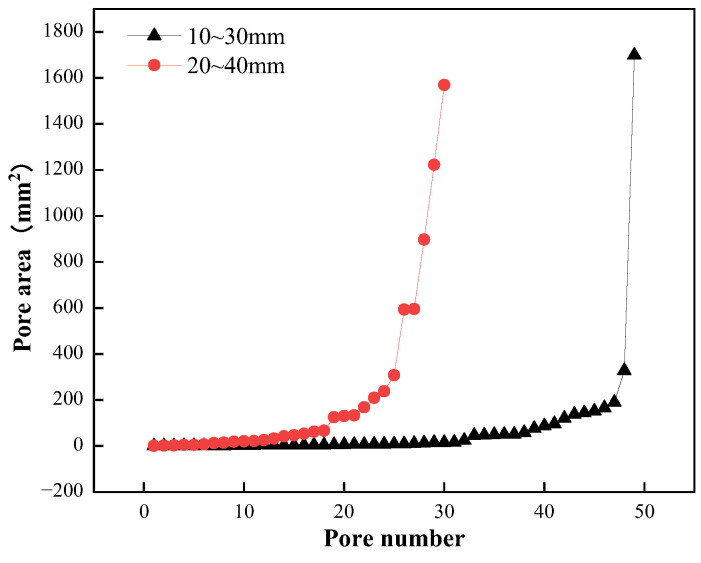
Pore area distribution of planting concrete planes.

The area of each pore in the plane of the planting concrete is shown in Figure 6. The abscissa is the slice pore number, which corresponds to the pore area on the ordinate. As shown in Figure 6, the distribution of porosity of planting concrete planes is similar in different aggregate sizes, with 16% porosity of planting concrete planes when the aggregate size is 10–30 mm, with a large number of planar pores and a pore area concentrated in the range of 0–200 mm^2^. At an aggregate size of 20–40 mm, the planar porosity of the planting concrete is 29%, with a low number of planar pores but the existence of large pores.

**Figure 7:**
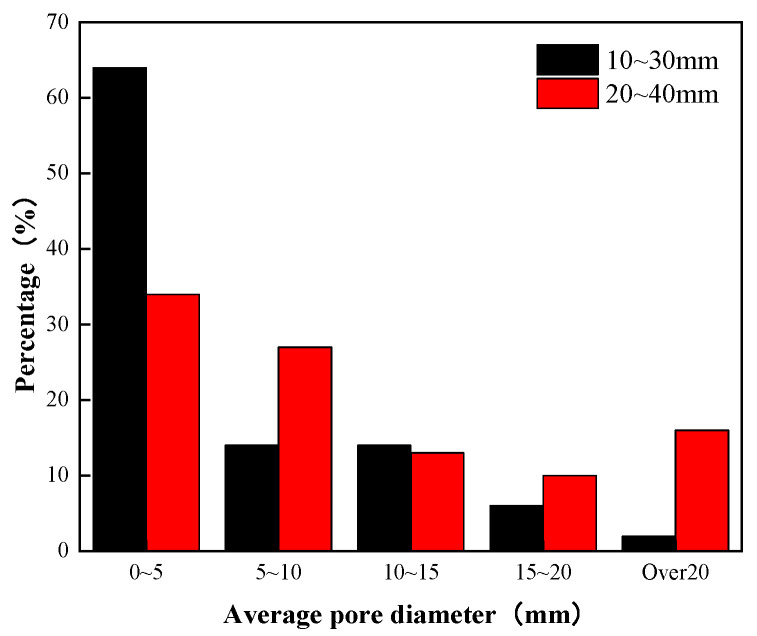
Average diameter distribution of pore spaces in planting concrete.

The average diameter distribution of the pore space in the plane of the planting concrete is shown in Figure 7. The average diameter reflects the general level of pore size of the specimen. As shown in Figure 7, planting concrete prepared with an aggregate particle diameter of 10–30 mm has 64% tiny pores with an average diameter below 5 mm and only 2% larger pores over 20 mm. This shows that the internal structure of the planting concrete prepared with 10–30 mm aggregates is denser and stronger. Planting concrete prepared from 20–40 mm aggregates has a more even distribution of average pore diameter, with only 34% tiny pores with an average pore diameter of less than 5 mm and 16% larger pores over 20 mm. This shows that the internal pores of the planting concrete prepared with 20–40 mm aggregate are mostly larger pores, which are conducive to the development of plant root systems.

**Figure 8:**
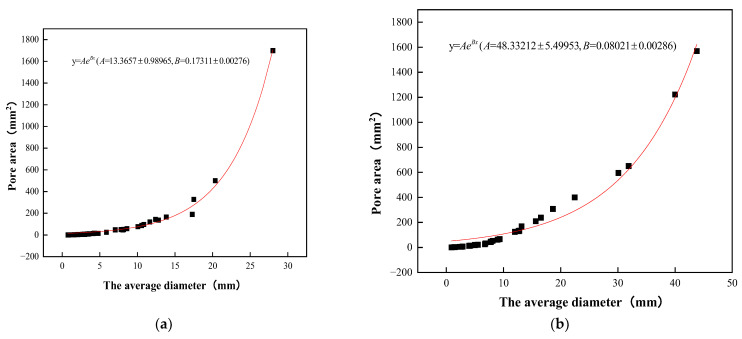
Average diameter in relation to planar pore area: (**a**) 10–30 aggregate gradation; (**b**) 20–40 aggregate gradation.

The average diameter of planting concrete versus planar pore area is shown in Figure 8. As shown in Figure 8, the average diameter of the planting concrete shows an exponential relationship with the porosity of the planes. The R^2^ of the exponential relationship equation $y=Ae^{bx}$ is 0.98. It shows a strong relationship between the average diameter of the pores and the area of the planar pores.

### 3.3. Effect of Water–Cement Ratio and Porosity on Compressive Strength

A reasonable porosity has a positive effect on plant growth, but porosity and compressive strength are in inverse proportion to each other. Therefore, it is necessary to study the relationship between water–cement ratio, porosity, and compressive strength at the same time.

**Figure 9:**
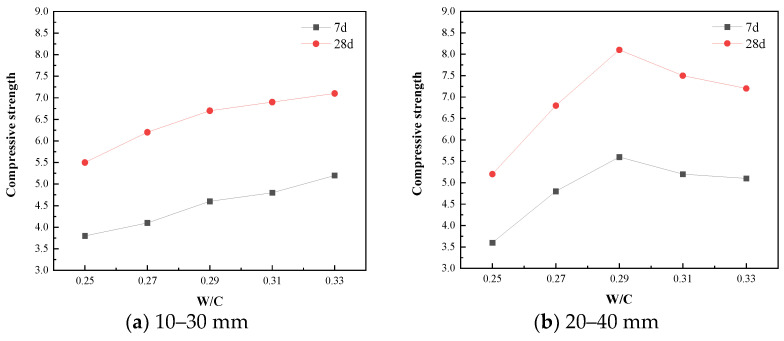
Relationship between compressive strength and water–cement ratio.

**Figure 10:**
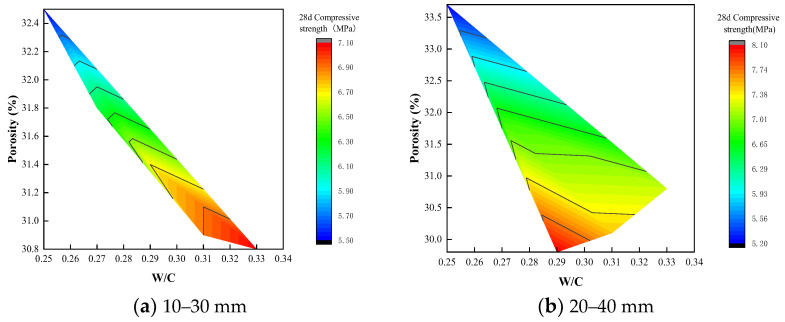
Effect of water–cement ratio and porosity on compressive strength.

Figure 9 is a graph showing the relationship between the compressive strength of the planting concrete and the water–cement ratio. It can be seen from Figure 9 that under the aggregate gradation of 10–30 mm, the compressive strength increases with the increase in the water–cement ratio, but under the aggregate gradation of 20–40 mm, the compressive strength first increases and then decreases. The effect of water–cement ratio and porosity on compressive strength is shown in Figure 10. As shown in Figure 10, with aggregate gradations of 10–30 mm, the optimum water–cement ratio should be controlled at 0.31–0.33, when the compressive strength ranges from 6.90 to 7.10 MPa and the porosity ranges from 30.8 to 31.0%. With aggregate gradations of 20–40 mm, the optimum water–cement ratio should be controlled at 0.28–0.30, when the compressive strength ranges from 7.75 to 8.10 MPa and the porosity ranges from 29.8 to 30.5%.

**Figure 11:**
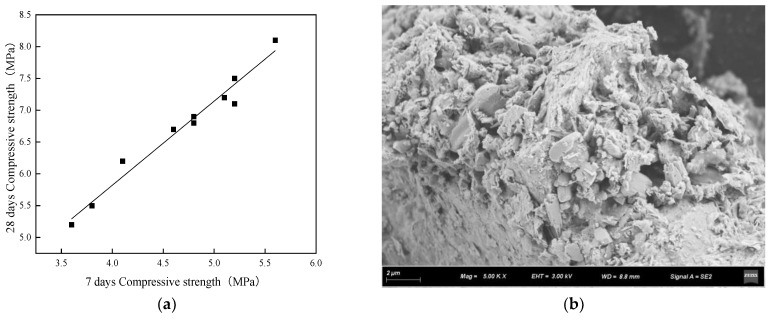
Compressive strength development and SEM images of planting concrete. (**a**) Fitting of curves. (**b**) SEM image of planting concrete (7 days).

The development of the compressive strength of the concrete is shown in Figure 11. As shown in Figure 11a, the compressive strength of the planting concrete grows quickly, and the 7-day strength of the planting concrete is approximately 66–75% of the 28-day strength. The regression analysis revealed a linear relationship between the 28-day compressive strength and the 7-day compressive strength, which is given in Equation (4).

(4) $$f_{c28}=1.549f_{c7}-0.657$$

This is due to the existence of a great number of pores within the planting concrete, which allows the cement and water to come into full contact at an early stage, and thus the hydration is adequate. As shown in Figure 11b, at the age of 7 days of planting concrete, the surface of the slurry is relatively rough, but its hydration products can be closely wrapped around the aggregate, and the internal material structure is basically continuous, without the formation of cracks and other defects.

### 3.4. Relationship between Compressive Strength and Porosity

**Figure 12:**
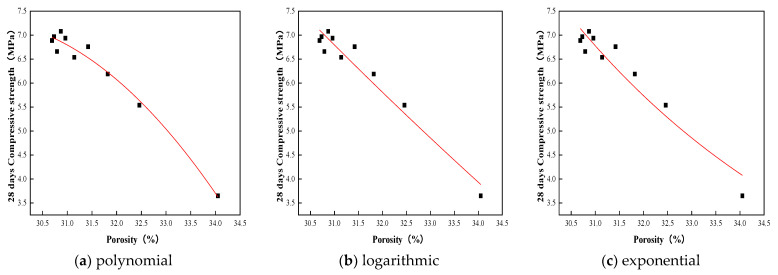
Fitting of curves for compressive strength versus porosity.

Experimental studies have been carried out on the relationship between porosity and strength of different porous materials. Hasselmann et al. [20] proposed a polynomial applicable to the relationship between porosity and strength of materials; Schiller et al. [21] proposed a logarithmic relationship to describe the relationship between porosity and strength of materials. Ryshkevitch et al. [22] proposed that the exponential relationship is preferable for describing the relationship between porosity and strength of porous materials. The exponential relationship proposed by Ryshkevitch was verified analytically by Chindaprasirt et al. [23,24] in their study, which proved to be valid for describing the relationship between porosity and strength of concrete. However, the porosity of planting concrete is considerably greater than that of ordinary concrete and can even reach 30%, while the strength is also smaller, at only 5–10 MPa. So, when considering the case of planting concrete, the effect of large connective pores is even more significant. Regression analysis was carried out for the test results of the planting concrete in this experiment. Three function types were selected for fitting to obtain the relationship between porosity and 28-day compressive strength; the regression analysis of the relationship is shown in Table 4, and the fitting of curves is shown in Figure 12. The specific regression analysis ideas were as follows: (1) establishing a scattering relationship between porosity and compressive strength; (2) reference to existing empirical models and selection of the type of relationship based on scatter plots; (3) using least squares to find the parameters; and (4) using the correlation test to determine the efficacy of the test formula. Comparing the three relational equations reflecting the strength of concrete and porosity, the exponential function relational equation $y={\mathrm{Ae}}^{\mathrm{Bx}}$ has an R^2^ of 0.97. This indicates that it is best at describing the correlation between porosity and strength and therefore expresses the relationship more appropriately. It shows that the model can effectively describe the relationship between the compressive strength and the porosity of the planting concrete.

### 3.5. Planting Experiment

**Figure 13:**
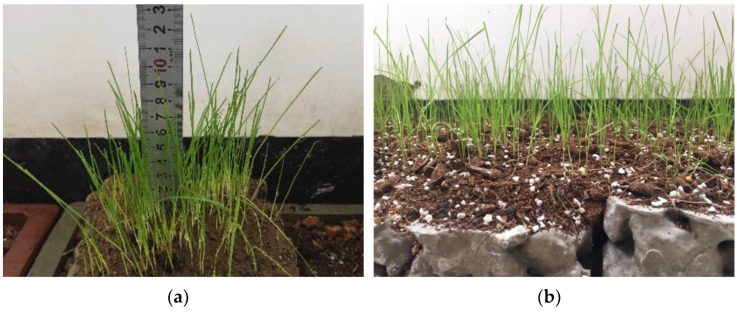
Ten-day growth. (**a**) Measuring plant height; (**b**) Plant growth.

**Figure 14:**
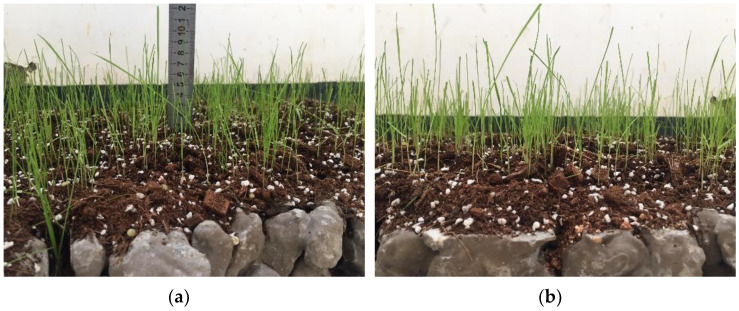
Twenty-five-day growth.(**a**) Measuring plant height; (**b**) Plant growth.

According to the results of the porosity and compressive strength tests, the porosity and compressive strength of the planting concrete specimens with 20–40 mm aggregate gradation and 0.29 water–cement ratio are 29.7% and 8.1 MPa, respectively, which can satisfy the needs of plant growth and are suitable for practical projects. Therefore, the planting concrete with these proportions was selected for the planting experiment, and the seeds were spread by hand at an amount of 30–40 g/m^2^ [25]. After sowing, a layer of loose soil 0.5–1 cm thick was used to cover the surface of the seeds and sprayed with water to avoid soil loss. All tall fescue seeds completed germination 3 days after sowing; plants were capable of reaching 3–4 cm in height after 4 days, and all plants were around 10 cm in height at 10 days. Plant growth of tall fescue at 10 days of sowing is shown in Figure 13. Tall fescue grows very quickly in 20 days, but the seedlings are brittle and easily fall over, and they can be kept upright by trimming the stems and leaves. After 20 days, tall fescue rhizomes are tougher, and the plants are primarily pruned to enable them to grow upright without limiting their height too much. While the upper area of the plant is growing well, the root system of tall fescue is developing very rapidly. When the plants had grown naturally to a height of 10 cm after 10 days, the root whiskers had reached 7 cm and could pass through a 5 cm thick test block of planting concrete; the root system was able to reach 12 cm in length at 25 days and penetrate a 10 cm thick planting concrete trial block. Plant growth at 25 days of sowing of tall fescue is shown in Figure 14. Although the pH of the planting concrete made of ordinary Portland cement is higher than that of the optimum growth environment for tall fescue, the mixing proportion design allows the planting concrete to meet the strength requirements with excellent porosity and an internal environment that meets the needs of plant growth.

### 3.6. Biomass and Elemental Content of Plant Leaves and Roots

Selected planting concrete specimens with the gradation of 20–40 mm and the water–cement ratio of 0.29 were sown with 4.5 g and 9 g, and the sowing method was mixed with surface sowing. Mixed planting consists of placing concrete blocks on the surface of the soil and then pouring soil and seeds with water into the holes of the concrete blocks; surface sowing consists of placing concrete blocks on the soil surface and then spreading the seeds on the surface over the holes in the concrete blocks. After sowing, the surface is covered with a layer of loose soil 0.5–1 cm thick and sprayed with water to avoid soil loss. After the planting is completed, daily watering is performed to ensure that the top and bottom of the concrete blocks are saturated with water.

**Figure 15:**
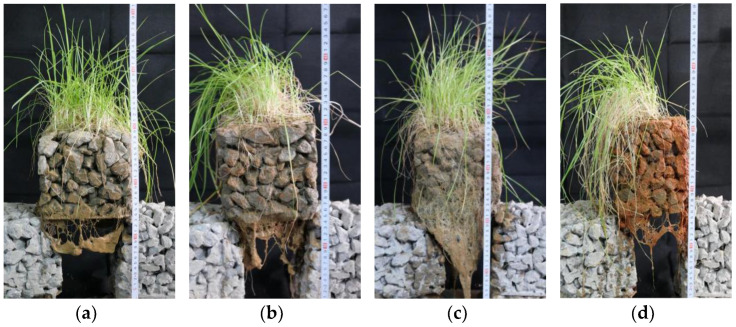
Fifty-day root system of tall fescue plant growth. (**a**) Mixed planting of 4.5 g seeds. (**b**) Mixed planting of 9 g seeds. (**c**) Surface planting of 4.5 g seeds. (**d**) Surface planting of 4.5 g seeds.

Figure 15 shows the root growth of tall fescue plants at 50 days after planting. Figure 15 shows the plant cover of different plant seed sowing methods corresponding to plant growth; there is a clear difference in the root system around and at the base of the concrete block, where the plant cover is approximately 85% for surface plantings and 50% for mixed plantings. By checking the root system, it was found that most of the roots reached the concrete pores, but the surface planting had more roots around and at the base of the planting concrete block. The experiment shows that surface planting is more effective than mixed planting. In addition, there were differences in plant cover and root systems around and at the base of concrete blocks for plants grown at different seed densities. Plant cover is approximately 85% for plants grown with 4.5 g of seeds mixed in and 45% for plants grown with 9 g of seeds; by checking the root system, it was found that most of the roots reached the concrete pores but the concrete block mixed with 4.5 g of seeds had more roots around and at the bottom. This is because proper seed density can alleviate or optimize plant competition for resources such as space, water, nutrients, and light [26,27].

**Figure 16:**
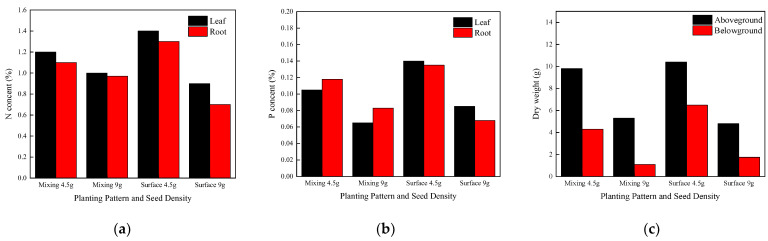
The elemental content and aboveground and belowground biomass of tall fescue. (**a**) Total nitrogen content. (**b**) Total phosphorus content. (**c**) Aboveground and belowground Biomass.

Figure 16 shows a graph of the elemental content of the leaves and roots of tall fescue with aboveground and belowground biomass. As shown in Figure 16, plant growth is influenced by the method of planting and the density of the seeds. Planting 4.5 g tall fescue seeds by surface planting has the highest leaf and root total nitrogen, total phosphorus, and aboveground and belowground biomass, with a good planting effect; planting 9 g tall fescue seeds by surface planting has the lowest total nitrogen content, lowest aboveground and belowground biomass, low total phosphorus content, and negative planting effect; with the mixed method of planting, the planting concrete sown with 4.5 g of tall fescue seeds planted was more effective than that sown with 9 g. Plant height, cover, number of individuals and root condition around and at the bottom of the concrete block, leaf rootstock element content, and aboveground and belowground biomass can be used as indicators to assess the performance of planting concrete implantation.

## 4. Conclusions

(1) The porosity of planting concrete decreases with increasing water–cement ratio, and the larger the aggregate particle size, the greater the porosity at the same water–cement ratio.
(2) With the same water–cement ratio, the porosity of the planting concrete prepared with 10–30 mm aggregates is lower than that of the planting concrete prepared with 20–40 mm aggregates. The average diameter of the planting concrete shows an exponential relationship with the porosity of the planes; the exponential relationship equation is $y=Ae^{Bx}$.
(3) With an aggregate gradation of 10–30 mm, the optimum water–cement ratio should be controlled at 0.31–0.33, when the compressive strength ranges from 6.90 to 7.10 MPa and porosity is 30.8–31.0%. With the aggregate gradation of 20–40 mm, the optimum water–cement ratio should be controlled at 0.28–0.30, at which point the compressive strength ranges from 7.75 to 8.10 MPa and the porosity ranges from 29.8 to 30.5%.
(4) The growth of the compressive strength of the planting concrete is rapid in the early stages, with a linear relationship between the 28-day compressive strength and the 7-day compressive strength: $f_{c28}=1.549f_{c7}-0.657$. A fitting analysis of the relationship between compressive strength and porosity of the planting concrete was carried out; the correlation coefficient R^2^ was calculated, and it was found that the exponential model $y={\mathrm{Ae}}^{\mathrm{Bx}}$ could better reflect the relationship between compressive strength and porosity of the planting concrete.
(5) Tall fescue can adapt to a degree of acidity and salinity and is a resilient growing plant, suitable for planting on planting concrete. The height of all 10-day plants was around 10 cm, the root system reached 7 cm in length, and the roots were entering the internal pores of the concrete. The roots of the 25-day plants reached a length of 12 cm and were able to puncture a 10 cm thick planting concrete test block.
(6) The effectiveness of planting concrete is influenced not only by its own properties, but also by the way in which the plants are planted and the density at which they are planted. The selection of surface planting methods with a reasonable seed density can achieve excellent planting results. In addition, plant height, cover, number of individuals and root condition around and at the base of the concrete block, leaf rootstock element content, and aboveground and belowground biomass can be used to assess the vegetation performance of the planting concrete.

## Figures and Tables

**Table 1:** Chemical composition of cement (%).

Cement Type	CaO	SiO_2_	Al_2_O_3_	Fe_2_O_3_	MgO	SO_3_	L.O.I
P O 42.5	65.52	22.45	4.49	4.13	1.47	0.94	1.09

**Table 2:** Physical properties of cement.

Cement Type	Specific Surface Area (m^2^ kg^−1^)	Density (g cm^−3^)	Water Consumption at Standard Consistency (%)	Setting Time (min)		Compressive Strength (MPa)		Flexural Strength (MPa)	
				Initial	Final	1 Day	7 Days	1 Day	7 Days
P O 42.5	368	3.1	25	185	240	9.6	34.5	1.9	6.1

**Table 3:** Physical properties of aggregates.

Particle Size (mm)	Surface Density (kg m^−3^)	Tight Packing Density (kg m^−3^)	Tight Packing Porosity (%)	Water Content (%)	Water Absorption (%)	Crushing Indicators(%)
10–30	2710	1520	44.3	1.5	0.35	11.0
20–40	2690	1565	41.8	3.1	0.92	13.7

**Table 4:** Regression analysis of the relationship between compressive strength and porosity.

Type	Equation	R^2^
Polynomial	y = A+ B_1_x + B_2_x^2^	0.92
Logarithmic function	y = Aln(x) + B	0.94
Exponential function	y = Ae^Bx^	0.97

Note: x: porosity; y: 28-day compressive strength; A, B_1_, B_2_, B are empirical constants.

## Data Availability

Not applicable.
